# Clairvoyant Melon Maturity Detection Enabled by Doctor-Blade-Coated Photonic Crystals

**DOI:** 10.3390/s21217046

**Published:** 2021-10-24

**Authors:** Yi-Cheng Lu, Liang-Cheng Pan, Yao-Wei Lei, Kun-Yi Andrew Lin, Hongta Yang

**Affiliations:** 1 Department of Chemical Engineering, National Chung Hsing University, 145 Xingda Road, Taichung City 40227, Taiwan; initial0210@gmail.com (Y.-C.L.); benny60422@gmail.com (L.-C.P.); wlo1630708@gmail.com (Y.-W.L.); 2 Department of Environmental Engineering, National Chung Hsing University, 145 Xingda Road, Taichung City 40227, Taiwan

**Keywords:** fruit maturity detection, ethanol vapor, photonic crystals, doctor-blade-coating, visual colorimetric readout

## Abstract

Climacteric fruits are harvested before they are ripened to avoid adverse damages during transport. The unripe fruits can undergo ripening processes associated with rind color changes on exposure to ethanol vapors. Although rind coloration is a common indicator showing fruit maturity, the attribute does not provide reliable assessment of maturity especially for melons. Herein, we report the achievement of sensitive and reversible melon maturity detection using macroporous hydrogel photonic crystals self-assembled by a roll-to-roll compatible doctor-blade-coating technology. The consumption of applied ethanol vapor during melon ripening results in less condensation of ethanol vapor in the pores (250 nm in diameter), leading to a distinct blue-shift of the optical stop band from 572 to 501 nm and an obvious visual colorimetric readout from yellow green to blue. Moreover, the dependence of the color change on Brix value within the melon has also been evaluated in the study.

## 1. Introduction

Fruits can be categorized into two types: non-climacteric fruits and climacteric fruits [1,2]. Non-climacteric fruits have to stay on plants to undergo ripening processes, which are associated with modifications in fruit composition, taste, texture, aroma and rind color [3]. Once harvesting, the fruits cannot gain sweetness and flavor anymore. As opposed to those, climacteric fruits, such as tomatoes, bananas, plums, peaches, melons and many others, are able to reach full physiological maturity even after all whole fruits are cut-up from plants [4,5,6]. Benefiting from the characteristic, immature climacteric fruits are harvested to extend shelf-life and to minimize adverse bruising during shipment. Afterwards, the management of fruit maturity can be implemented by introducing exogenously applied ethanol, which promotes ripening of climacteric fruits [7,8,9]. Depending on the fruit cultivar and the fruit maturity, different amounts of ethanol vapor are applied to accelerate converting starch into sugar within fruits. As a result, starchy, crunchy and bland tasting climacteric fruits are softened and gain more flavors in the ripening processes. In addition to that, the fruit ripening is accompanied with a series of biochemical and physiological changes, including decrease in organic acids, fruit texture softening, formation of volatile chemical compounds, non-photosynthetic pigment accumulation, and degradation of chlorophyll [10,11].

Determining the best moment for tasting fruits, which influences the commercial acceptability, is utmost important for fresh fruit marketing. Among a variety of fruit maturity detecting indicators, rind coloration is the most common one showing fruit maturity [12]. Take an example, bananas turn from green to yellow, and display brown speckling on rinds as they reach optimum maturity. Recently, a fruit maturity index related to the chlorophyll amount presented in fruit is developed by assessing the index of the absorption difference (IAD index), where the IAD index decreases during fruit ripening [13,14,15]. The promising tool provides a platform to monitor fruit maturity and to establish an optimal fruit marketing timing. However, this attribute cannot offer reliable assessments of maturity for a wide range of fruits without distinguishable rind color changes during ripening, such as melons. Fruit texture, primarily affected by fruit softening, is another important attribute to determine fruit maturity. The physical and mechanical textural characteristics, associated with alterations of polysaccharide components in cell walls, cellular turgor and changes in cuticle architecture, can be evaluated by instrumental and sensory assessments. The evaluations are capable of providing qualitative and quantitative information on flesh melting, crispiness, crunchiness, and firmness, which are utilized to determine fruit maturity [16,17,18,19,20,21]. Unfortunately, either instrumental or sensory methodologies require considerable effort and time, limiting their practical applications. Besides fruit textures, many other indicators, e.g., Brix value, total acidity and soluble solids concentration, have also been studied to detect fruit maturity [22,23,24]. Although sophisticated instrumentation is not highly required, their assessments suffer from laborious operating procedure, time-consuming analysis and inevitable fruit destruction. Consequently, there is an urgent demand to propose a rapid, straightforward, and non-destructive methodology for on-site fruit maturity detection.

Inspired by the spectacular colors on peacock feathers, Tropical Morpho butterfly wings, beetle shells, and cuttlefish skins, photonic crystals have been widely investigated [25,26,27,28]. The photonic band gap materials, consisting of periodic dielectric structures, bring about forbidden energy gaps for commensurate wavelengths of light. By monitoring the color changes of the diffractive media though altering the effective refractive indices and the lattice spacings under chemical or biochemical stimulations, the amounts of selected chemicals or biomaterials can be deduced. On account of the distinct optical characteristics, numerous photonic crystal-based chemical and biological detectors with visual readouts have been designed in recent years [29,30,31,32,33,34]. Nevertheless, most photonic crystals manufactured by lithography-based approaches are impeded by complex and time-consuming fabrication procedures using sophisticated equipment [35,36]. In comparison to those approaches, spontaneous crystallization of monodisperse colloids, induced by electric fields, magnetic fields, gravity force, surface tensions, capillary forces, can serve as templates to manufacture photonic crystals [37,38,39,40,41]. Even though the self-assembly approaches are simple and inexpensive, the methodologies are only accessible for low-throughput production. Moreover, it still remains a challenge to develop clairvoyant fruit maturity detectors based on the tunable structural colors.

To address the issues, we show that three-dimensional macroporous hydrogel photonic crystals developed by integrating ethanol-sensitive polymers and a roll-to-roll compatible self-assembly technology enable on-site detection of fruit maturity. Take muskmelon as an example, the consumption of applied ethanol vapor during muskmelon ripening leads to less condensation of ethanol vapor in the pores, resulting in a highly visual readout. Importantly, the clairvoyant melon maturity detector is portable, inexpensive and fast responsive for practical applications.

## 2. Materials and Methods

### 2.1. Chemicals and Solvents

The reagents utilized for silica colloid synthesis, including tetraethyl orthosilicate (≥99 wt.%), ethanol (200 proof), and aqueous ammonium hydroxide (30–33 wt.%), are provided by Merck KGaA. Ethoxylated trimethylolpropane triacrylate (ETPTA, SR-454) monomers and 2-hydroxyethyl methacrylate (HEMA) (≥99 wt.%) monomers are acquired from Sartomer Americas and Merck KGaA, respectively. Initiators, 2-hydroxy-2-methyl-1-phenyl-1-propanone (97 wt.%) and azobisisobutyronitrile (98 wt.%) are purchased from Merck KGaA. Aqueous hydrofluoric acid (≥48 vol.%), used to wet etch silica colloids, is collected from Merck KGaA. All chemicals and solvents are of analytical reagent quality and applied directly without any purification. Ultrapure water is obtained from a LABSTAC WU113 laboratory ultrapure water system. The unripe muskmelons with green rinds, tough blossom-ends, and stems still attached, are picked and provided by Taiwan Fresh Fruit Marketing Cooperative.

### 2.2. Colloidal Self-Assembly by Doctor-Blade-Coating

After purification with 200 proof ethanol, monodispersed StÖber silica colloids are dispersed in ETPTA monomers with 2-hydroxy-2-methyl-1-phenyl-1-propanone (1 vol.%) as a photoinitiator [42]. The volume fraction of silica colloids is controlled to be 74 vol.%. The colloidal suspension is then doctor-blade-coated on a poly(ETPTA) coated glass substrate using a modified Proyes PFA-2010-S automatic film applicator with a constant coating speed of 1 cm/min. In the coating procedure, the blade provides a one-dimensional shear force to align the silica colloids. After all, the ETPTA monomers are photopolymerized by exposure to ultraviolet radiation for 5 s (X Lite 500 Pulsed curing system, OPAS, Portland, OR, USA).

### 2.3. Preparation of Macroporous Poly(HEMA)/Poly(ETPTA) Photonic Crystals

The templating silica colloids can be selectively wet-etched by dropping a diluted hydrofluoric acid aqueous solution (1 vol.%) onto the silica colloidal crystal/poly(ETPTA) composite, followed by rinsing with ultrapure water. The resulting macroporous poly(ETPTA) photonic crystals are immersed in a mixture of HEMA monomers (20 vol.%), azobisisobutyronitrile (1 vol.%), and ethanol (84 vol.%), followed by spinning the photonic crystals at 500 rpm for 2 min (WS-400B-6NPP-Lite, Laurell, North Wales, PA, USA) to eliminate redundant mixture retained on the surface. After ethanol evaporation, the HEMA monomers are polymerized at 70 °C to fabricate macroporous poly(HEMA)/poly(ETPTA) photonic crystals.

### 2.4. Experimental Procedures for Muskmelon Maturity Sensing

The free-standing macroporous poly(HEMA)/poly(ETPTA) photonic crystals and an unripe muskmelon are placed in a sealed chamber, which is evacuated and subsequently aerated with demanded ethanol vapor at 25 °C. The chamber is finally backfilled with air to maintain a constant pressure of 1 atm. Normal-incidence optical reflection spectra of the macroporous photonic crystals under various ethanol vapor partial pressures are evaluated using an optical fiber probe sealed in the chamber using a silicon-based high vacuum leak sealant (AGB, Agar Scientific, Stansted, Essex, UK). A bubble testing is performed by immersing the sealed chamber in a water-filled test container to provide indications of the existence of leaks.

### 2.5. Determination of Brix Values in Muskmelons

After peeling and removing seeds, the muskmelon meat is mashed and centrifuged at 5000 rpm for 20 min. The sugar content (Brix) of the resulting supernatant is determined using a digital Brix-meter (Atago PR-1 Refractometer, Taichung, Taiwan). The results are conducted by averaging 20 measurements on each muskmelon.

### 2.6. Characterization

Scanning electron microscopy (SEM) is carried out on a JEOL 7001F FE-SEM. The specimens are coated with gold/palladium alloy (Cressington 108 Auto sputter coater, Chalk Hill, Watford, UK) prior to imaging. Photographic images are collected from a Nikon Z50 digital camera. Normal incidence optical reflection spectra in the wavelength range from 300 to 800 nm are performed using an Ocean Optics HR4000 UV-vis-near-IR spectrometer with an Ocean Optics DT-MINI-2-GS deuterium tungsten halogen light source and an Ocean Optics R400-UV-VIS optical fiber (wavelength range: 300 nm to 1.1 μm).

## 3. Results

**Figure 1:**
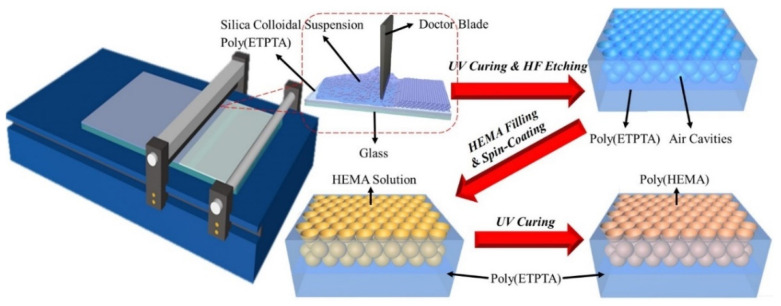
Schematic illustration of the experimental procedures for fabricating large-area macroporous photonic crystals.

The fabrication process of macroporous photonic crystal-based fruit maturity sensors is schematically illustrated in Figure 1. In brief, a mixture of monodispersed silica colloids and ETPTA monomers is deposited onto a poly(ETPTA) coated glass substrate. The silica colloids are shear-aligned in a scalable doctor-blade-coating procedure, followed by a UV-triggered polymerization procedure to engineer silica colloidal crystal/poly(ETPTA) composites. After wet-etching the embedded silica colloids, the resulting macroporous poly(ETPTA) photonic crystals are peeled off and immersed in an ethanol-based HEMA monomer mixture. The HEMA monomers can finally be polymerized at 70 °C to engineer macroporous poly(HEMA)/poly(ETPTA) photonic crystals.

**Figure 2:**
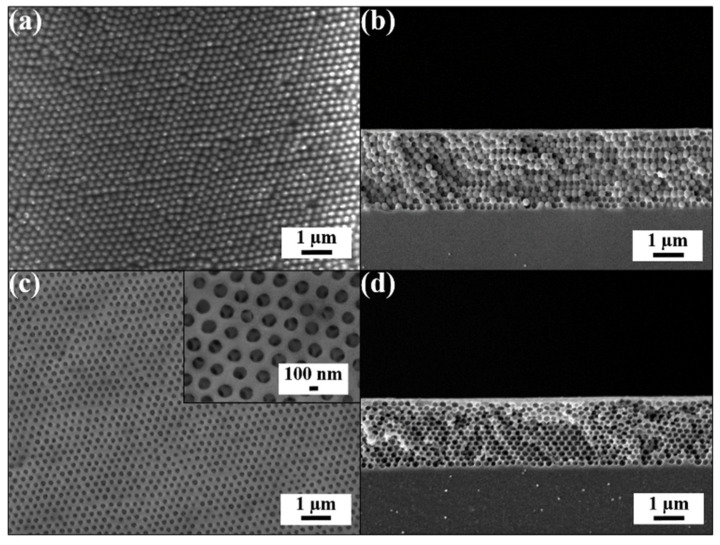
(**a**) Top-view SEM image of a doctor-blade-coated silica colloidal crystal/poly(ETPTA) composite consisting of 250 nm silica colloids. (**b**) Cross-sectional SEM image of the specimen in (**a**). (**c**) Top-view SEM image of macroporus poly(ETPTA) photonic crystals templated from 250 nm silica colloidal crystals. The insert displays a magnified top-view SEM image. (**d**) Cross-sectional SEM image of the specimen in (**c**).

The three-dimensionally long-range hexagonal ordering of 250 nm silica colloids is verified by SEM images of the doctor-blade-coated silica colloidal crystal/poly(ETPTA) composite (Figure 2a,b). The embedded close-packed silica colloids can be completely wet-etched to fabricate free-standing macroporous poly(ETPTA) photonic crystals (Figure 2c,d). It is noteworthy that the protrusion depth of top-layer silica colloids from the poly(ETPTA) matrix is shallower than the silica colloid radius, leading to the non-close-packed appearance of top-layer pores. Indeed, the three-dimensionally close-packed macroporous structures are well-retained during the wet-etching procedure.

**Figure 3:**
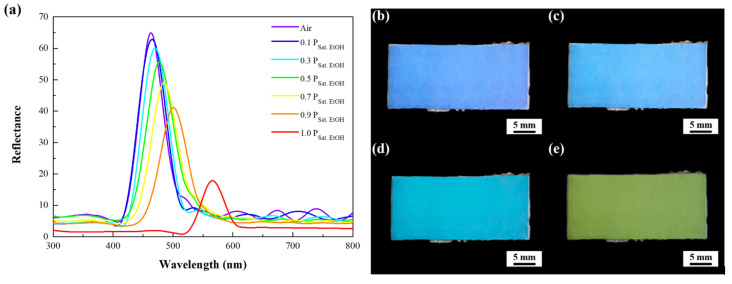
(**a**) Normal-incidence optical reflection spectra acquired from macroporous poly(ETPTA) photonic crystals templated from 250 nm silica colloidal crystals under various ethanol vapor partial pressures. Photographic images of the macroporous photonic crystals at (**b**) 0 P_Sat. EtOH_, (**c**) 0.3 P_Sat. EtOH_, (**d**) 0.7 P_Sat. EtOH_ and (**e**) 1.0 P_Sat. EtOH_. P_Sat. EtOH_ represents the saturated ethanol vapor pressure at 25 °C.

The diffraction gratings constructed of three-dimensionally close-packed 250 nm pores are capable of featuring an iridescent color. The structural coloration originates from the interference between multiple reflections from the parallel pore arrays as visible light travels by different paths in the photonic crystals. In other words, iridescence is created when part of incident light is reflected from the top surfaces of the pores, while a further part of the rest light penetrated through them is reflected from their bottom surfaces. The reflected waves travel back upward in the same direction and superpose to form a resultant wave of greater amplitude controlled by the thickness and refractive index of the pores. To evaluate optical characteristics of the doctor-blade-coated macroporous poly(ETPTA) photonic crystals templated from 250 nm silica colloidal crystals, the spectral reflection at normal incidence is measured in dry air environment (Figure 3a). It is noticed that the reflection peak position of the photonic crystals locates at 463 nm, which agrees well with the calculated value (464 nm) estimated using Bragg’s equation:

(1) $$\lambda_{peak}=2\cdot n_{eff\;}\cdot d$$

in which $n_{eff}$ and *d* represent the effective refractive index and the lattice spacing of the as-prepared photonic crystals, respectively [43]. The effective refractive index can be further expressed as:

(2) $$n_{eff}=n_{poly\left(ETPTA\right)}\cdot VF_{poly\left(ETPTA\right)}+n_{air}\cdot VF_{air}$$

where $n_{poly\left(ETPTA\right)}=1.46$ and $n_{air}=1$. The volume fractions of poly(ETPTA) ($VF_{poly\left(ETPTA\right)}$) and air ($VF_{air}$) in macroporous photonic crystals equal to 0.26 and 0.74, respectively. This agreement evidences that the pores are three-dimensionally close-packed. Fruit maturity can be determined by assessing the presence of ethanol vapor partial pressure in the environment during fruit ripening. According to that, visible ethanol vapor sensing characteristics of the macroporous poly(ETPTA) photonic crystals is investigated under various ethanol vapor partial pressures at ambient conditions (25 °C and 1 atm) to evaluate their melon maturity detection capability. The condensation of ethanol vapor in the pores creates a higher effective refractive index of medium, and therefore results in a red-shift of reflection peak. It is found that the reflection peak position shifts from 463 to 565 nm when the ethanol vapor partial pressure increases from 0 P_Sat. EtOH_ to 1.0 P_Sat. EtOH_, where P_Sat. EtOH_ denotes the saturation vapor pressure of ethanol at 25 °C. As a result, the corresponding appearance of the photonic crystals changes from blue to yellow green with the increase of ethanol vapor partial pressures (Figure 3b–e). More specifically, the photonic crystals are able to exhibit various colors under different melon maturity levels.

## 4. Discussion

**Figure 4:**
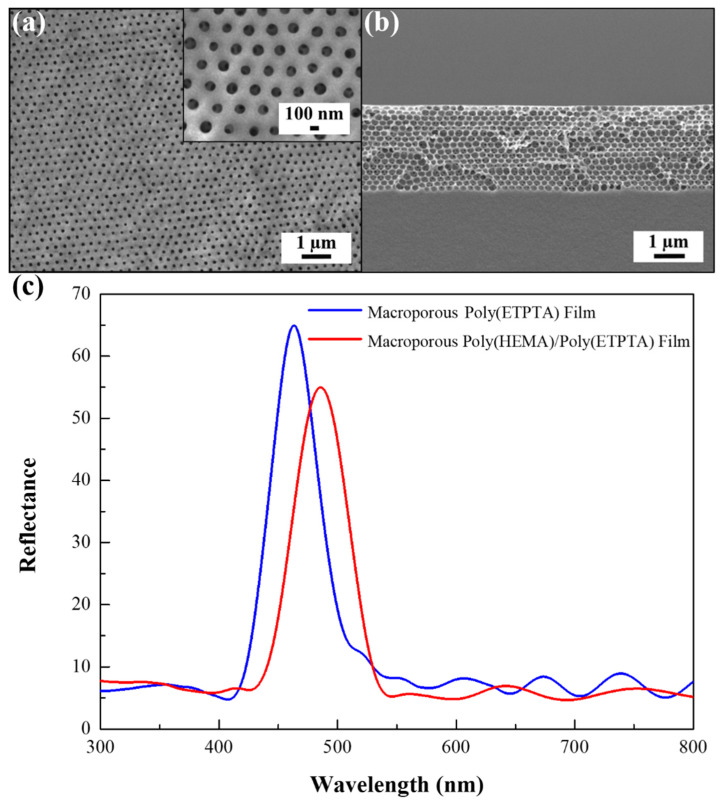
(**a**) Top-view SEM image of macroporus poly(HEMA)/poly(ETPTA) photonic crystals templated from 250 nm silica colloidal crystals. The insert displays a magnified top-view SEM image. (**b**) Cross-sectional SEM image of the specimen in (**a**). (**c**) Normal-incidence optical reflection spectra acquired from the macroporous poly(ETPTA) photonic crystals and the corresponding macroporous poly(HEMA)/poly(ETPTA) photonic crystals.

In order to further improve the sensitivity of melon maturity detection, the poly(ETPTA) pores are uniformly coated with poly(HEMA), which is highly responsive to ethanol vapor, for fabricating macroporous poly(HEMA)/poly(ETPTA) photonic crystals. In comparison with the pores of poly(ETPTA) photonic crystals, a decrease in top-layer pore opening and an increase in wall thickness are evident on the poly(HEMA)/poly(ETPTA) photonic crystals (Figure 4a,b). It is worth mentioning that the coating layer results in a higher $n_{eff}$ and the corresponding reflection peak position therefore shifts to 486 nm (Figure 4c). The poly(HEMA) layer thickness can be calculated using the above-mentioned Bragg’s equation. Here,

(3) $$n_{eff}=n_{poly\left(ETPTA\right)}\cdot VF_{poly\left(ETPTA\right)}+n_{poly\left(HEMA\right)}\cdot VF_{poly\left(HEMA\right)}+n_{air}\cdot VF_{air}\;$$

In the equation, $n_{poly\left(HEMA\right)}$ equals to 1.45, while $VF_{poly\left(HEMA\right)}$ can be expressed as $0.74\;-\;VF_{air}$. The computed results indicate that the volume fraction of poly(HEMA) in the pores is 19 vol.% ($VF_{poly\left(HEMA\right)}$ = 0.14), which agrees well with the volume fraction of HEMA (20 vol.%) in the coating mixture. In addition, it is found that the poly(HEMA) coating layer thickness is around 10 nm.

**Figure 5:**
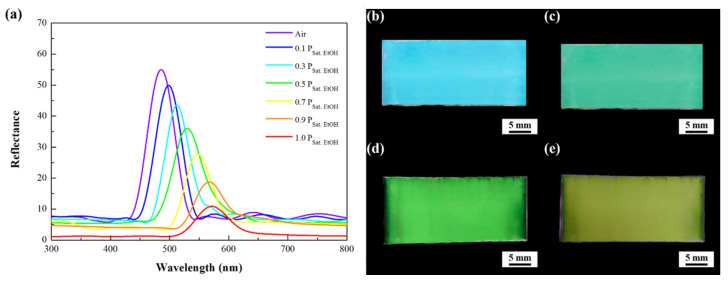
(**a**) Normal-incidence optical reflection spectra acquired from macroporous poly(HEMA)/poly(ETPTA) photonic crystals templated from 250 nm silica colloidal crystals under various ethanol vapor partial pressures. Photographic images of the macroporous photonic crystals at (**b**) 0 P_Sat. EtOH_, (**c**) 0.3 P_Sat. EtOH_, (**d**) 0.7 P_Sat. EtOH_ and (**e**) 1.0 P_Sat. EtOH._ P_Sat. EtOH_ represents the saturated ethanol vapor pressure at 25 °C.

**Figure 6:**
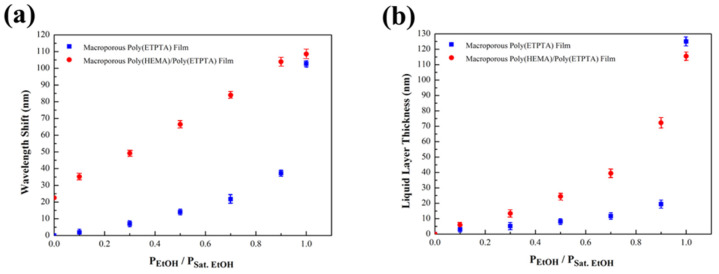
(**a**) The reflection peak position shifts of the macroporous photonic crystals templated from 250 nm silica colloidal crystals under various ethanol vapor partial pressures. (**b**) Calculated condensed ethanol layer thicknesses at various ethanol vapor partial pressures. P_EtOH_ and P_Sat. EtOH_ represent the actual ethanol vapor partial pressure and the saturated ethanol vapor pressure at 25 °C, respectively.

The sensitivity of ethanol vapor detection for the as-fabricated macroporous poly(HEMA)/poly(ETPTA) photonic crystals is assessed by collecting optical reflection spectra of the photonic crystals under various ethanol vapor partial pressures. It is found that the reflection peak red-shifts with the increase of ethanol vapor partial pressures, actuating a corresponding color change (Figure 5). Importantly, the reflection peaks of macroporous poly(HEMA)/poly(ETPTA) photonic crystals display larger wavelength shifts than those of macroporous poly(ETPTA) photonic crystals on exposure to ethanol vapor (Figure 6a). The results can be expounded by the Flory-Huggins free energy theory [44]. On account of a favorable Gibbs free energy change accompanying mixing poly(HEMA) with ethanol, poly(HEMA) behaves a high swelling degree in the presence of ethanol. As a result, ethanol vapor is apt to condense in the macroporous poly(HEMA)/poly(ETPTA) photonic crystals, leading to a higher effective refractive index and a larger red-shift in reflection peak. It is noteworthy that the red-shift increases linearly with ethanol vapor partial pressure, which is critical in melon maturity detection. The linear optical response against vapor partial pressure can again be explicated using Bragg’s equation, in which

(4) $$n_{eff}=n_{poly\left(ETPTA\right)}\cdot VF_{poly\left(ETPTA\right)}+n_{poly\left(HEMA\right)}\cdot VF_{poly\left(HEMA\right)}+n_{air}\cdot VF_{air}+n_{liquid\;ethanol}\cdot VF_{liquid\;ethanol}\;$$

By presuming on the ethanol vapor condenses in the pores uniformly, $VF_{poly\left(ETPTA\right)}$, $VF_{poly\left(HEMA\right)}$ and $VF_{air}$ can be regarded as 0.26, 0.14 and (1 − 0.26 − 0.14$\;-\;VF_{liquid\;ethanol}$), respectively. The computed volume fraction of liquid ethanol ($VF_{liquid\;ethanol}$) is further applied to calculate the condensed liquid ethanol layer thickness (Figure 6b). Clearly, thicker condensed liquid ethanol layer associated with higher ethanol vapor partial pressures are demonstrated. Moreover, the difference in calculated layer thickness between the macroporous poly(HEMA)/poly(ETPTA) photonic crystals and the macroporous poly(ETPTA) photonic crystals is greater under higher vapor partial pressure. The results can be interpreted using Kelvin equation:

(5) $$ln\frac{P_{EtOH}}{P_{Sat.\;\;Etoh}}=\frac{2\cdot V_{m}\cdot \gamma }{r\cdot R\cdot T}$$

where $V_{m}$, γ, r, R and T denote the molar volume of liquid ethanol, the surface tension of liquid ethanol, the radius of the resulting air cavity, the ideal gas constant, and the absolute temperature, respectively. With constant $V_{m}$, γ, R and T, $ln\frac{P_{EtOH}}{P_{Sat.\;Etoh}}$ is proportional to $\frac{1}{r}$. In other words, thicker liquid ethanol layers are created in the pores under higher ethanol vapor partial pressures. The greater ethanol vapor condensation further generates a higher swelling degree of poly(HEMA) layer, resulting in a difference in wavelength shift between the macroporous poly(HEMA)/poly(ETPTA) photonic crystals and the macroporous poly(ETPTA) photonic crystals. Consequently, the sensitivity of photonic crystal-based ethanol vapor sensing is improved by introducing a poly(HEMA) coating layer.

**Figure 7:**
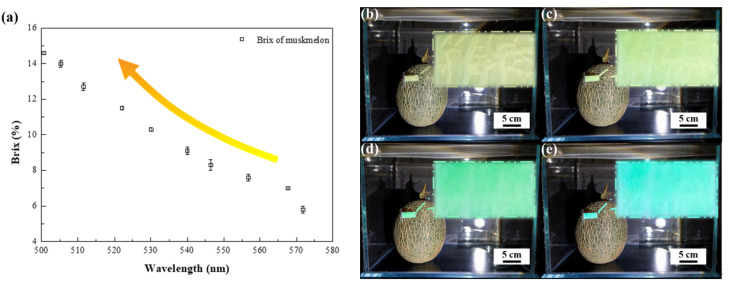
(**a**) Dependence of the Brix value in a muskmelon on the reflection peak position of the macroporous poly(HEMA)/poly(ETPTA) photonic crystals templated from 250 nm silica colloidal crystals as the muskmelon is exposed to 1.00 P_Sat. EtOH_. Photographic images of the muskmelon and the macroporous photonic crystals at (**b**) day 1, (**c**) day 3, (**d**) day 6 and (**e**) day 9.

The melon maturity detecting capability of the macroporous poly(HEMA)/poly(ETPTA) photonic crystals is further verified at ambient conditions. To recognize an appropriate initial ethanol vapor partial pressure in muskmelon ripening, each unripe muskmelon (~500 g in average) is individually placed in a sealed glass chamber (30 L), on the inner wall of which is dispensed a piece of free-standing macroporous photonic crystals. After evacuation, the chambers are aerated with demanded ethanol vapor, and then backfilled with air to maintain a constant pressure of 1 atm. The muskmelons of each treatment are sampled for every 12 h to evaluate their maturities, which are associated with pigment changes and sugar releases within the muskmelons. It is obvious that the muskmelon ripening hastened by ethanol leads to a fast and drastic change in the accumulation of sugar content (Brix) (Figure S1). For untreated muskmelons, it takes about 15 days to reach full maturity. In comparison to that, the Brix of the saturated ethanol vapor-treated muskmelons achieves a highest value in 10 days. Importantly, the muskmelon tissues can convert applied ethanol to acetaldehyde, which leads to an increased conversion of 1-aminocyclopropane-1 carboxylic acid (ACC) to ethylene within the tissues. The production of ethylene causes denaturation of enzymes, accelerating tissue softening and converting starch into sugar. As a result, the muskmelon ripening is further promoted on exposure to higher ethanol vapor pressures. Although muskmelons gradually turn from greenish white to creamy yellow in rind background color during ripening, it is hard to judge with naked eyes (Figure S2). To express the rind background color change in a more standardized way, the Commission Internationale de L’Eclairage (CIE) chromaticity values of the saturated ethanol vapor-treated muskmelons at day 1, day 3, day 5, day 7 and day 9 are depicted in Figure S3. The resulting CIE chromaticity diagram indicates that the rind colors are virtually indistinguishable from each other. In contrast, the macroporous poly(HEMA)/poly(ETPTA) photonic crystals in the chamber are capable of exhibiting a distinct color change with varying muskmelon maturities. The consumption of applied ethanol vapor during muskmelon ripening causes less condensation of ethanol vapor in the pores, leading to a lower effective refractive index of medium (Figure S1). As a result, the reflection peak blue-shifts with the increases of Brix value (Figure 7a). It appears that the corresponding appearance of the macroporous photonic crystals changes from yellow green to blue (Figure 7b–e). It is worth noting that the lattice spacing of the macroporous photonic crystals can be adjusted to improve the sensitivity of muskmelon maturity detection. Macroporous photonic crystals templated from larger colloids behave larger blue-shifts on ethanol vapor sensing, which can easily monitor the muskmelon maturity from the dramatic color changing of the photonic crystals. Importantly, the as-developed on-site melon maturity detection method exhibits comparable sensitivity to existing detection methods, which suffer from sophisticated instrumentations, time-consuming analysis, and inevitable fruit destruction. (Table 1). In addition, the as-engineered clairvoyant melon maturity detector displaying a highly visible readout is small, portable and readily responsive.

## 5. Conclusions

In conclusion, three-dimensional macroporous poly(HEMA)/poly(ETPTA) photonic crystals are engineered through integrating a scalable doctor blade coating technology and a templating methodology. Owing to a favorable Gibbs free energy change on mixing poly(HEMA) with ethanol, poly(HEMA) coating layer behaves a high swelling degree in the presence of ethanol. Benefiting from that, the macroporous photonic crystals templated from 250 nm colloids exhibit a large stop band shift from 572 to 501 nm on exposure to varied ethanol vapor. The consumption of applied ethanol vapor during muskmelon ripening therefore leads to a distinct change in color on the photonic crystals. It is evidenced that the appearance of the photonic crystals turns from yellow green to blue as the Brix value within the muskmelon increases from 5.8 to 14.3%. This indicates that the as-fabricated macroporous photonic crystals display a highly visible readout for clairvoyant muskmelon maturity detection. We believe that the macroporous poly(ETPTA) scaffold can be coated with a variety of polymers with matching solubility parameters to specific chemicals, providing a universal route for chemical/biological sensing, pH detection, temperature monitoring, etc., without applying any instrumentation or label.

## Figures and Tables

**Table 1:** Summary of technical details of all melon maturity detection methods discussed in the article.

Detection Method	Instruments	Sensitivity	Reference
Regulation of Ethylene	MCP Treatments	2.5–5 (μL/L)	[16]
Brix Analysis	Refractometer	0–15.5 (°Brix)	[45]
Aroma Analysis	GC-MS/GC-FID	0–89/0–97 (%)	[45]
Brix Analysis	Refractometer	0–14.6 (°Brix)	[17]
Sugar Contents Analysis	HPLC	0–20 (mg/g)	[46]
Volatile Compounds Analysis	GC-MS	0–88.8 (%)	[18]
Volatile Compounds Analysis	GC–MS/GC-O-MS	0–93/0–77 (%)	[47]
Firmness Analysis	Firmness Tester	0–70 (N)	[19]
Texture Analysis	Texture Analyzer	0–16 (N)	[6]
Texture Evaluation	Texture Analyzer	0–1.0 (N)	[20]
Rind Color Analysis	Reflectance Meter	0.1–5.0	[21]

## Data Availability

The data presented in this study are available on request from the corresponding author.

## Supplementary Materials

The following are available online at https://www.mdpi.com/article/10.3390/s21217046/s1. Figure S1. Changes of Brix values with time of the muskmelons exposed to various ethanol vapor partial pressures. Figure S2. Photographic images of the muskmelons exposed to air (1st row), 0.32 P_Sat. EtOH_ (2nd row), 0.82 P_Sat. EtOH_ (3rd row), and 1.00 P_Sat. EtOH_ (4th row). Figure S3. The color coordinates of the muskmelon images, while the muskmelon is exposed to 1.00 P_Sat. EtOH_ for 1 day, 3 days, 5 days, 7 days, and 9 days, based on the CIE 1931 color space. Figure S4. Normal-incidence optical reflection spectra acquired from macroporous poly(HEMA)/poly(ETPTA) photonic crystals templated from 250 nm silica colloidal crystals as the muskmelon is exposed to 1.00 P_Sat. EtOH_.
