# Supplementary material for: Clairvoyant Melon Maturity Detection Enabled by Doctor-Blade-Coated Photonic Crystals
Source: Sensors (Basel). 2021 Oct 24;21(21):7046. doi: 10.3390/s21217046 (PMC8586947; doi:10.3390/s21217046)
Supplement: Supplementary file 1 [file sensors-21-07046-s001.zip › sensors-1404976-supplementary.pdf]

Clairvoyant Melon Maturity Detection Enabled by Doctor-Blade-Coated Photonic Crystals

Yi-Cheng Lu [1], Liang-Cheng Pan [1], Yao-Wei Lei [1], Kun-Yi Andrew Lin [2]*, and Hongta Yang [1]*

[1]Department of Chemical Engineering, National Chung Hsing University, 145 Xingda Road, Taichung City 40227, Taiwan
[2]Department of Environmental Engineering, National Chung Hsing University, 145 Xingda Road, Taichung City 40227, Taiwan
*Corresponding Author: hyang@dragon.nchu.edu.tw (H. Y.); linky@nchu.edu.tw (K.-Y. Lin)

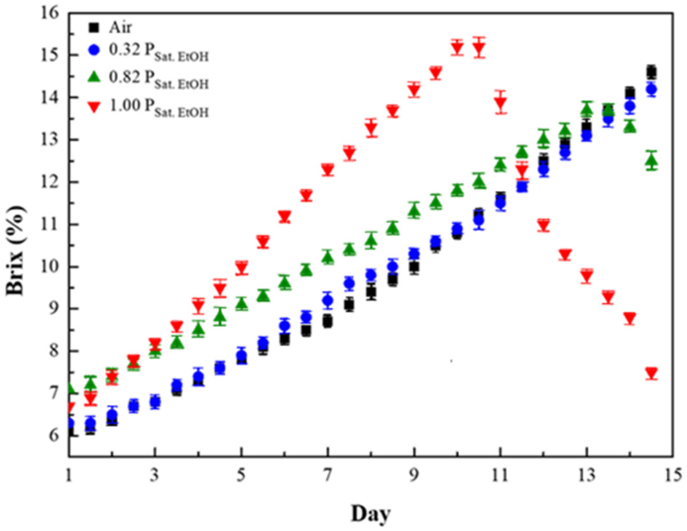

Figure S1. Changes of Brix values with time of the muskmelons exposed to various ethanol vapor partial pressures.

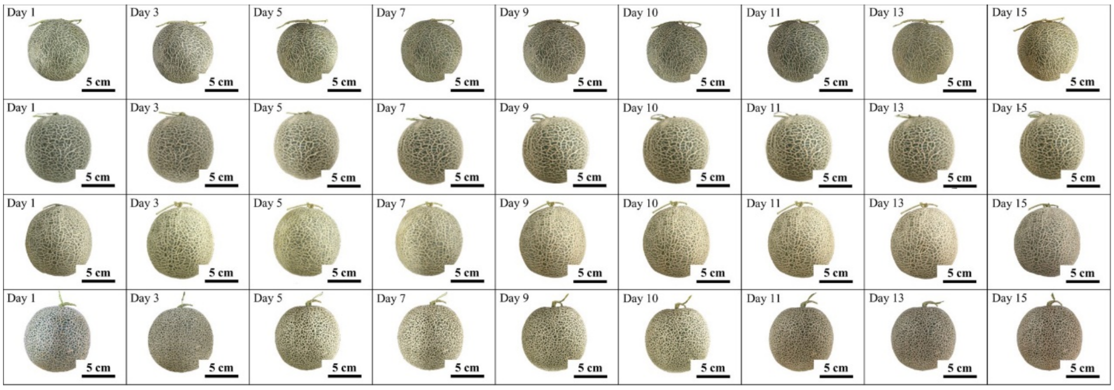

Figure S2. Photographic images of the muskmelons exposed to air (1st row), 0.32 $P_{Sat.\ EtOH}$ (2nd row), 0.82 $P_{Sat.\ EtOH}$ (3rd row), and 1.00 $P_{Sat.\ EtOH}$ (4th row).

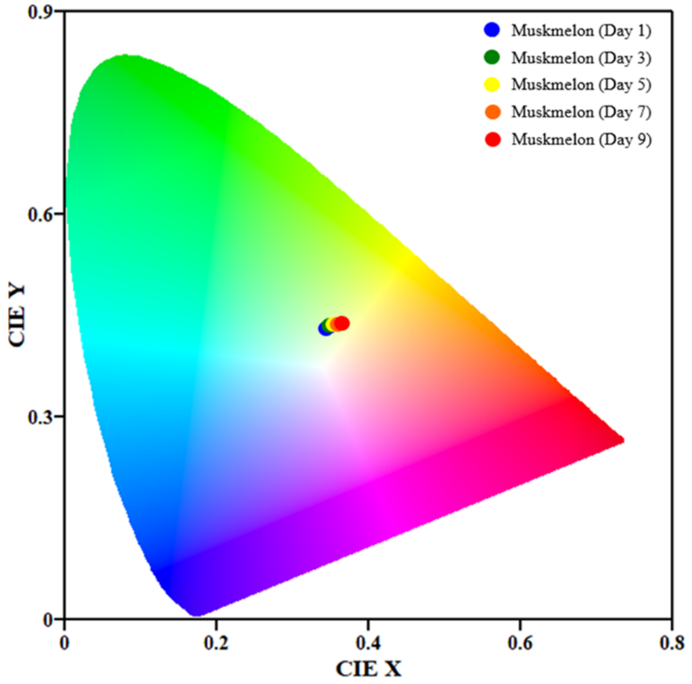

Figure S3. The color coordinates of the muskmelon images, while the muskmelon is exposed to 1.00 $P_{Sat.\ EtOH}$ for 1 day, 3 days, 5 days, 7 days, and 9 days, based on the CIE 1931 color space.

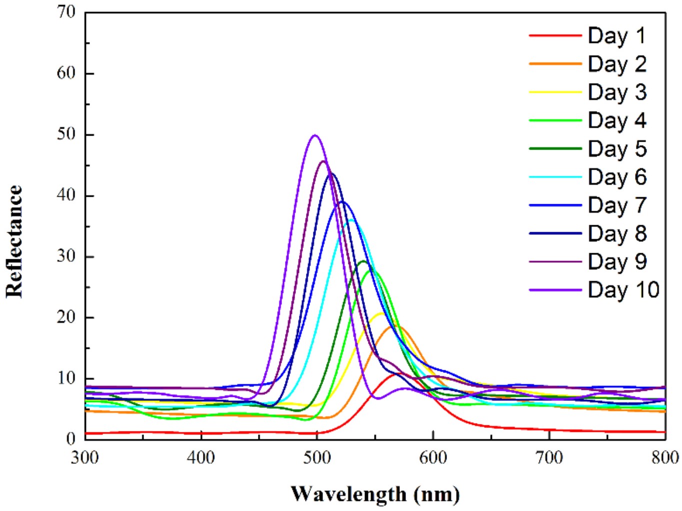

Figure S4. Normal-incidence optical reflection spectra acquired from macroporous poly(HEMA)/poly(ETPTA) photonic crystals templated from 250 nm silica colloidal crystals as the muskmelon is exposed to 1.00 $P_{Sat.\ EtOH}$.
